# Characteristics and outcomes of patients screened by rapid response team who transferred to the intensive care unit

**DOI:** 10.1186/s12873-022-00575-y

**Published:** 2022-02-03

**Authors:** Song-I. Lee, Jeong Suk Koh, Yoon Joo Kim, Da Hyun Kang, Jeong Eun Lee

**Affiliations:** Department of Pulmonary and Critical Care Medicine, Chungnam National University School of Medicine, 33 Munhwa-ro, Jung-gu, 301-721 Daejeon, Republic of Korea

**Keywords:** General ward, Intensive care unit, Prognosis, Rapid response team

## Abstract

**Background:**

The utilization of a rapid response team (RRT) has influenced the clinical outcomes of patients in the general ward. However, the characteristics of RRT-screened patients who are transferred to the intensive care unit (ICU) are unknown. Therefore, the present study aimed to evaluate these factors.

**Methods:**

We conducted a retrospective study using patient data from a tertiary medical center in Republic of Korea between January 2016 and December 2017. Multivariate logistic regression analyses were performed to assess the factors associated with the risk of in-hospital mortality.

**Results:**

A total of 1,096 patients were included: 389 patients were transferred to the ICU, and 707 patients stayed in the ward. Patients in the ICU group were more likely to be admitted for medical reasons, hepatobiliary disease, and high heart rate. More interventions were performed, hospital stays were longer, and the 28-day and in-hospital mortality rates were higher in the ICU group than in the ward group. Multivariate logistic regression analyses showed that risk factors affecting ICU admission were higher Sequential Organ Failure Assessment (SOFA) score, National Early Warning Score (NEWS), platelet count, and lactate level. ICU transfer was not associated with in-hospital mortality.

**Conclusions:**

Among RRT-screened patients, those with higher SOFA score, NEWS, and lactate level were more likely to be transferred to the ICU. Therefore, these patients should be closely monitored and considered for ICU transfer.

## Background

A rapid response for general ward patients suffering from acute deterioration may be impossible because of missing symptoms and unrecorded vital signs. However, regular monitoring and introduction of automatic alarm systems aid the emergency medical team, which operates 24 h or part-time a day, to implement treatment and reduce mortality [1–3]. General ward patients (up to 10% of cases) experience unexpected events [4], and 7.3% of them experience fatal events [5]. Rapid response team (RRT) activation is usually triggered by several factors, such as monitoring of vital signs, pre-rounding, and direct calls from attending physicians, nurses, and family members [3, 5].

However, some studies have shown that having an RRT in the hospital is associated with higher intensive care unit (ICU) admissions and fewer severe patient transfer from the ward [6]. In addition, RRT intervention does not improve the disease severity and outcomes of patients transferred from the ward [6, 7]. These studies have usually compared the characteristics before and after the RRT intervention. Patients transferred to the ICU after RRT screening, and prognostic factors for RRT-screened patients are not well known. Thus, the present study investigated the characteristics and outcomes of patients who were transferred to the ICU among patients screened by RRT.

## Methods

### Study design and patient selection

This was a retrospective observational study of patients admitted to Chungnam National University Hospital, a 1200-bed tertiary academic hospital in South Korea, between January 2016 and December 2017. Patients with a Do Not Resuscitate (DNR) record were excluded because they affected the availability of interventions such as invasive mechanical ventilation and admission to the ICU [8–10].

### Rapid response team

We started operating an RRT at our hospital for adult patients from 7 AM to 11 PM daily on weekdays in 2014. The RRT consists of 3 ICU staff (ICU) and three dedicated nurses with experience in critical care. At least one intensivist and one dedicated nurse were on duty every day. The RRT was equipped for monitoring and resuscitation of airway, breathing, and circulatory emergency (patient monitor, emergency drugs, videolaryngoscope, point-of-care-testing arterial blood gas analysis, portable ultrasonography, and portable ventilator).

We screened adult patients over 18 years of age, excluding pediatric patients. Elderly and obstetric patients were included in the screening. Screening criteria from electronic medical records (EMRs) were as follows: systolic blood pressure ≤ 80 mmHg, respiratory distress (rate ≥ 30 breaths/min), saturation of percutaneous oxygen (SpO_2_ ≤ 85%), sudden mental change, or unexplained agitation. Admitted patients’ vital signs were checked regularly by nurses in the ward on every duty (every 8 h). If the patient in the ward was deteriorating, then vital signs were checked and recorded more frequently at 10-min to 1-h intervals. In addition, the EMR-based National Early Warning Score (NEWS) system was established in November 2013 and updated in December 2017, and it is used for adult patients admitted to the general ward [11]. The NEWS system is based on six physiological parameters: respiration rate, oxygen saturation, systolic blood pressure, pulse rate, level of consciousness or new confusion, and temperature. When a nurse in duty puts the above six parameters on the vital sign sheet of EMR, the score is automatically calculated. When the nurses of the ward recorded the vital signs in real time, if there was a vital sign abnormality or NEWS of more than 5 points [12], then the patient list and abnormal findings could be checked by RRT nurses.

The RRT screening was performed when 1) the measurements of a patient exceeded the pre-defined thresholds in the EMR-based automatic screening system, 2) doctors or nurses called the RRT for aid, or 3) code blue was announced for cardiopulmonary arrest.

ICU admission for patients screened at the RRT was decided by the intensivist in charge of the RRT at that time. Even when the RRT did not screen, the same intensivist decided whether to admit the ICU.

### Data collection

All study data were retrieved from the EMR (C&U Care, Daejeon, Republic of Korea). A total of 1,218 patients were screened by RRT between February 1, 2016, and December 31, 2017. The study enrolled 1096 patients, excluding 122 patients with DNR. Demographic, clinical, and radiological information, as well as laboratory and imaging data, was collected. Interventions performed at RRT included those performed at the ward and those performed within 24 h of admission to the ICU after deciding to be performed at the RRT.

Sequential Organ Failure Assessment (SOFA) scores based on Vincent et al. were used to predict mortality during the first 24 h of ICU admission [13]. The worst value was chosen for each organ system every 24 h to calculate the score [13]. The NEWS system was used for making acute illness assessments and determining responses [14]. The reason for RRT screening was defined by referring to the test findings of the patients at the time of screening for RRT and the patient's vital signs and patient’s history.

Ethics approval.

This study was approved by the Institutional Review Board (IRB) (IRB No: CNUH 2019–06-030), and the requirement for informed consent was waived because of the retrospective nature of the study.

### Statistical analysis

All values are expressed as mean ± standard deviation and median and interquartile ranges for continuous variables and percentages for categorical variables. Student’s t-test or the Mann–Whitney U test was used for continuous data, and Pearson’s chi-square test or Fisher’s exact test was used for categorical data analysis. Predictors of disease severity were identified through univariate logistic regression analysis. Multivariate logistic regression analyses with a backward elimination procedure, including all predictors with a p-value of ≤ 0.05 in the univariate analysis, were performed to obtain the adjusted odds ratio (OR) along with 95% confidence interval (CI) and to determine the variables independently associated with disease severity. All *p*-values were two-tailed, and p-values of < 0.05 were considered statistically significant. All statistical analyses were performed using SPSS software (version 22.0; IBM Corporation, Somers, NY, USA).

## Results

### Patients’ baseline characteristics

**Fig. 1:**
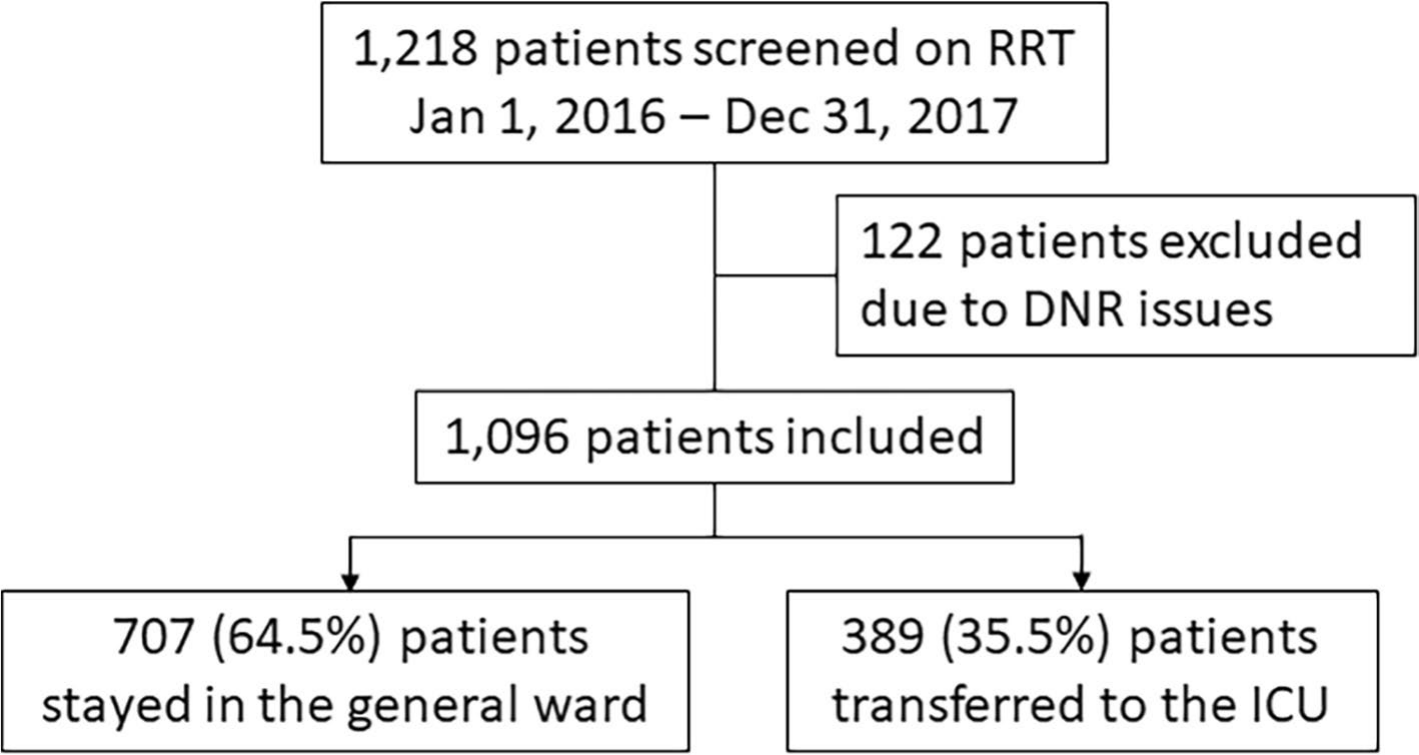
Flowchart of the patient selection process. RRT, rapid response team; DNR, do not resuscitate; ICU, intensive care unit

Of the total 1096 enrolled patients, 707 (64.5%) stayed in the ward and 389 (35.5%) were transferred to the ICU (Fig. 1). The characteristics of the 1,096 patients are presented in Table 1. More patients were admitted for medical reasons (75.8% vs. 70.3%, *p* = 0.050) among those admitted to the ICU (ICU group). There were fewer cases of chronic lung disease (9.3% vs. 15.4%, *p* = 0.004), and chronic hepatobiliary disease (12.1% vs. 7.8%, *p* = 0.019) was the more common underlying disease in the ICU group. There were no significant differences in other underlying diseases.

**Table 1:** Characteristics of the included patients

	All patients (*n* = 1096)	Ward group (*n* = 707)	ICU group (*n* = 389)	*P*-value
Age	67.4 ± 14.7	67.8 ± 14.8	66.5 ± 14.6	0.145
Male	675 (61.6)	428 (60.5)	247 (63.5)	0.335
BMI	22.5 ± 4.4	22.5 ± 4.4	22.7 ± 4.3	0.551
Medical	792 (72.3)	497 (70.3)	295 (75.8)	0.050
Surgical	304 (27.7)	210 (29.7)	94 (24.2)	0.050
Underlying disease				
Solid tumor	182 (16.6)	123 (17.4)	59 (15.2)	0.342
Hematologic malignancy	54 (4.9)	41 (5.8)	13 (3.3)	0.072
Chronic lung disease	145 (13.2)	109 (15.4)	36 (9.3)	0.004
Chronic heart disease	231 (21.1)	145 (20.5)	86 (22.1)	0.535
Chronic hepatobiliary disease	102 (9.3)	55 (7.8)	47 (12.1)	0.019
Cerebrovascular disease	182 (16.6)	120 (17.0)	62 (15.9)	0.660
Chronic kidney disease	116 (10.6)	68 (9.6)	48 (12.3)	0.161
Diabetes	339 (30.9)	213 (30.1)	126 (32.4)	0.438
Transplantation	3 (0.3)	2 (0.3)	1 (0.3)	0.938
Most recent vital sign^a^				
MBP, mmHg	86.7 (3.3 – 102.2)	86.7 (73.3 – 100.0)	86.7 (71.7 – 103.3)	0.568
HR, bpm	103 (88 – 120)	102 (88 – 116)	106 (90 – 124)	0.002
RR, /min	24 (20 – 30)	24 (20 – 29)	24 (20 – 30)	0.325
BT, ℃	37.4 (36.8 – 38.0)	37.4 (36.9 – 38.0)	37.4 (36.8 – 38.0)	0.238
SpO2, %	95 (91 – 98)	95 (92 – 98)	95 (91 – 98)	0.238
Laboratory findings				
WBC, × 10^3^/uL	10.38 (7.19 – 14.40)	9.98 (7.1 – 13.8)	10.8 (7.5 – 16.0)	0.100
Hb, g/dL	10.3 (9.0 – 12.0)	10.3 (9.0 – 12.1)	10.1 (8.8 – 11.8)	0.448
Platelet, × 10^3^/uL	182 (116 – 261)	192 (123 – 266)	170 (101 – 250)	0.018
T-bilirubin, mg/dL	0.71 (0.48 – 1.27)	0.70 (0.45 – 1.19)	0.80 (0.50 – 1.40)	0.018
Creatinine, mg/dL	0.85 (0.62 – 1.47)	0.78 (0.59 – 1.24)	0.97 (0.71 – 1.90)	0.002
Lactate, mEq/L	1.8 (1.1 – 3.1)	1.6 (1.1 – 2.5)	2.2 (1.2 – 3.8)	0.213
CRP, mg/dL	8.8 (2.9 – 16.5)	8.5 (2.9 – 15.7)	9.0 (2.8 – 17.6)	< 0.001
SOFA score	4 (2 – 6)	3 (2 – 5)	5 (3 – 7)	< 0.001
NEWS score	8 (6 – 10)	7 (6 – 9)	8 (6 – 10)	< 0.001
Hospitalization period prior to RRT activation (Days)	5 (1 – 15)	4 (1 – 14)	6 (1 – 17)	0.268

Data are presented as mean (SD), n (%), or median (interquartile range), unless otherwise indicated

^a^It was a vital sign at the time of screening for RRT in the patient and when RRT was activated

*ICU* Intensive care unit, *BMI* Body mass index, *MBP* Mean blood pressure, *HR* Heart rate, *RR* Respiratory rate, *BT* Body temperature, *SpO*_*2*_ Saturation by pulse oximetry, *SD* Standard deviation, *WBC* White blood cell, *Hb* Hemoglobin, *CRP* C-reactive protein, *SOFA* Sequential Organ Failure Assessment, *NEWS* National Early Warning Score, *RRT* Rapid response team

Vital signs recorded when the RRT first screened the patient showed that the heart rate was faster in the ICU group (106 [90–124] vs. 102 [88–116], beats/min, *p* = 0.002). Laboratory findings showed that the platelet count was lower (170 [101–250)]vs. 192 [123–266], × 10^3^/µL, *p* = 0.018), and T-bilirubin [0.80 (0.50–1.40) vs. 0.70 (0.45–1.19), mg/dL, *p* = 0.018], creatinine (0.97 [0.71–1.90] vs. 0.78 [0.59–1.24], mg/dL, *p* = 0.002), and C-reactive protein (CRP) levels (9.0 [2.8–17.6] vs. 8.5 [2.9–15.7] mg/dL, *p* < 0.001) were higher in the ICU group. The SOFA score (5 [3–7] vs. 3 [2–5], *p* < 0.001) and NEWS (8 [6–10] vs. 7 [6–9], *p* < 0.001) were higher in the ICU group. Other vital signs and laboratory findings showed no significant differences between groups (Table 1).

### Intervention and Outcomes

Table 2 showed the interventions and outcomes implemented after the RRT screening. Reasons for screening by RRT were sepsis, which accounted for fewer cases, and septic shock, respiratory distress, and cardiogenic shock, which accounted for more cases, in the ICU group. RRT interventions were more frequent in the ICU group, except for extracorporeal membrane oxygenation, renal replacement therapy, application of high-flow nasal cannula, and ultrasonography. Hospital length of stay (LOS) was longer (33 [17–70] vs. 25 [13–47], *p* = 0.011), and 28 day-mortality (22.6% vs. 14.6%, *p* = 0.001) and in-hospital mortality (29.8% vs. 20.1%, *p* < 0.001) were higher in the ICU group.

**Table 2:** Reasons for screening by RRT, intervention, and outcomes

	All patients (*n* = 1096)	Ward group (*n* = 707)	ICU group (*n* = 389)	*P*-value
Reason for screening by RRT				
Sepsis	834 (76.1)	606 (85.7)	228 (58.6)	< 0.001
Septic shock	126 (11.5)	48 (6.8)	78 (20.1)	< 0.001
Respiratory distress	85 (7.8)	39 (5.5)	46 (11.8)	< 0.001
Cardiogenic shock	51 (4.7)	14 (2.0)	37 (9.5)	< 0.001
RRT intervention				
ACLS	41 (3.7)	9 (1.3)	32 (8.2)	< 0.001
ECMO	3 (0.3)	1 (0.1)	2 (0.5)	0.258
Renal replacement therapy	17 (1.6)	10 (1.4)	7 (1.8)	0.622
Intubation	142 (13.0)	13 (1.8)	129 (33.2)	< 0.001
Ventilator	49 (4.5)	6 (0.8)	43 (11.1)	< 0.001
HFNC	124 (11.3)	99 (14.0)	25 (6.4)	< 0.001
A-line insertion	14 (1.3)	3 (0.4)	11 (2.8)	0.001
C-line insertion	35 (3.2)	6 (0.8)	29 (7.5)	< 0.001
USG	100 (9.1)	64 (9.1)	36 (9.3)	0.911
Vasopressors	83 (7.6)	22 (3.1)	61 (15.7)	< 0.001
Outcomes				
Hospital LOS	28 (14 – 56)	25 (13 – 47)	33 (17 – 70)	0.011
28 day mortality	191 (17.4)	103 (14.6)	88 (22.6)	0.001
In-hospital mortality	258 (23.5)	142 (20.1)	116 (29.8)	< 0.001

Data are presented as median (interquartile range) or number (%), unless otherwise indicated

*RRT* Rapid response team, *ICU* Intensive care unit, *ACLS* Advanced cardiovascular life support, *ECMO* Extracorporeal membrane oxygenation, *HFNC* High-flow nasal cannula, *A-line* Arterial line, *C-line* Central line, *USG* Ultrasonography, *LOS* Length of stay

### Factors associated with transfer to ICU

Multivariate analysis revealed factors associated with ICU transfer (Table 3). After adjusting for confounders, the independent predictors of ICU transfer included SOFA score (OR, 1.281; 95% CI, 1.184–1.386; *p* < 0.001), NEWS (OR, 1.065; 95% CI, 1.006–1.128; *p* = 0.032), platelet count (OR, 1.002; 95% CI, 1.001–1.004; *p* = 0.002), and lactate level (OR, 1.161; 95% CI, 1.074–1.255; *p* < 0.001).

**Table 3:** Multivariate logistic regression analysis of factors associated with transfer to the ICU

	Univariate analysis			Multivariate analysis		
	OR	95% CI	*P*-value	OR	95% CI	*P*-value
Age	0.994	0.986 – 1.002	0.146			
Male	1.134	0.878 – 1.464	0.335			
BMI	1.009	0.979 – 1.040	0.551			
Medical	1.326	1.000 – 1.759	0.050	1.045	0.731 – 1.493	0.811
SOFA score	1.297	1.226 – 1.371	< 0.001	1.281	1.184 – 1.386	< 0.001
NEWS	1.117	1.068 – 1.167	< 0.001	1.065	1.006 – 1.128	0.032
Vital sign						
MBP	1.002	0.996 – 1.008	0.547			
HR	1.009	1.003 – 1.014	0.001	1.005	0.997 – 1.012	0.210
RR	1.010	0.991 – 1.029	0.301			
Underlying disease						
Solid tumor	0.849	0.605 – 1.191	0.343			
Hematologic malignancy	0.562	0.297 – 1.061	0.076			
Chronic lung disease	0.560	0.375 – 0.834	0.004	0.742	0.458 – 1.202	0.225
Chronic heart disease	1.100	0.814 – 1.487	0.535			
Chronic hepatobiliary disease	1.629	1.080 – 2.457	0.020	1.189	0.708 – 1.998	0.512
Cerebrovascular disease	0.927	0.663 – 1.297	0.660			
Chronic kidney disease	1.323	0.894 – 1.958	0.162			
Diabetes	1.111	0.851 – 1.450	0.438			
Laboratory findings						
WBC	1.010	0.997 – 1.022	0.128			
Hb	0.977	0.923 – 1.034	0.426			
Platelet	0.999	0.998 – 1.000	0.018	1.002	1.001 – 1.004	0.002
T-bilirubin	1.047	1.005 – 1.090	0.026	0.975	0.936 – 1.016	0.224
Creatinine	1.115	1.037 – 1.198	0.003	0.918	0.828 – 1.018	0.104
Lactate	1.251	1.164 – 1.345	< 0.001	1.161	1.074 – 1.255	< 0.001
CRP	1.009	0.995 – 1.022	0.213			

*ICU* Intensive care unit, *OR* Odds ratio, *CI* Confidence interval, *BMI* Body mass index, *SOFA* Sequential Organ Failure Assessment, *NEWS* National Early Warning Score, *MBP* Mean blood pressure, *HR* Heart rate, *RR* Respiratory rate, *WBC* White blood cell, *Hb* Hemoglobin, *CRP* C-reactive protein

Factors associated with patients’ in-hospital mortality.

Multivariate analysis revealed factors associated with in-hospital mortality (Table 4). The independent predictors of in-hospital mortality included age (OR, 1.029; 95% CI, 1.013–1.045; *p* < 0.009), screened due to medical reason (OR, 1.799; 95% CI, 1.115–2.904; *p* = 0.016), SOFA (OR, 1.119; 95% CI, 1.042–1.202; *p* = 0.002), solid tumor (OR, 1.676; 95% CI, 1.065–2.638; *p* = 0.026), hematologic malignancy (OR, 3.166; 95% CI, 1.483–6.760; *p* = 0.003), total bilirubin (T-bilirubin; OR, 1.055; 95% CI, 1.006–1.106; *p* = 0.027), lactate level (OR, 1.166; 95% CI, 1.078–1.261; *p* < 0.001), and CRP level (OR, 1.027; 95% CI, 1.008–1.046; *p* = 0.005) after adjusting for confounders.

**Table 4:** Multivariate logistic regression analysis of factors associated with in-hospital mortality

	Univariate analysis			Multivariate analysis		
	OR	95% CI	*P*-value	OR	95% CI	*P*-value
Age	1.020	1.010 – 1.031	< 0.001	1.029	1.013 – 1.045	< 0.001
Male	1.246	0.931 – 1.668	0.140			
BMI	1.015	0.982 – 1.050	0.376			
Medical	2.262	1.580 – 3.237	< 0.001	1.799	1.115 – 2.904	0.016
SOFA score	1.235	1.169 – 1.305	< 0.001	1.119	1.042 – 1.202	0.002
NEWS	1.139	1.083 – 1.197	< 0.001	1.064	0.996 – 1.138	0.067
Underlying disease						
Solid tumor	2.075	1.474 – 2.923	< 0.001	1.676	1.065 – 2.638	0.026
Hematologic malignancy	2.993	1.719 – 5.211	< 0.001	3.166	1.483 – 6.760	0.003
Chronic lung disease	1.333	0.901 – 1.973	0.150			
Chronic heart disease	1.252	0.899 – 1.745	0.184			
Chronic hepatobiliary disease	1.475	0.943 – 2.307	0.088			
Cerebrovascular disease	0.934	0.639 – 1.365	0.724			
Chronic kidney disease	1.271	0.824 – 1.961	0.278			
Diabetes	0.981	0.725 – 1.328	0.902			
Laboratory findings						
WBC	1.004	0.993 – 1.016	0.477			
Hb	0.911	0.853 – 0.973	0.006	1.001	0.919 – 1.091	0.974
Platelet	0.997	0.995 – 0.998	< 0.001	0.999	0.3997 – 1.001	0.223
T-bilirubin	1.106	1.049 – 1.166	< 0.001	1.055	1.006 – 1.106	0.027
Creatinine	1.040	0.963 – 1.123	0.314			
Lactate	1.252	1.173 – 1.338	< 0.001	1.166	1.078 – 1.261	< 0.001
CRP	1.029	1.014 – 1.044	< 0.001	1.027	1.008 – 1.046	0.005
Transfer to the ICU	1.691	1.272 – 2.247	< 0.001	1.042	0.704 – 1.542	0.836

*OR* Odds ratio, *CI* Confidence interval, *BMI* Body mass index, *SOFA* Sequential Organ Failure Assessment, *NEWS* National Early Warning Score, *WBC* White blood cell, *Hb* Hemoglobin, *T-bilirubin* Total bilirubin, *CRP* C-reactive protein, *ICU I*ntensive care unit

## Discussion

In this study, 35.5% of the RRT-screened patients were transferred to the ICU. Patients admitted for medical reasons with an underlying chronic hepatobiliary disease or a higher SOFA score or NEWS were more likely to be admitted to the ICU when screened by RRT. Patients admitted to the ICU had a longer hospital LOS and higher 28-day mortality and in-hospital mortality rates. Higher SOFA, NEWS, platelet count, and lactate level were associated with ICU transfer. ICU transfer was not associated with in-hospital mortality.

RRT has been implemented in several hospitals to facilitate early recognition and treatment of deteriorating patients in wards [3, 15]. Most RRT activation leads to one or more interventions in patients, including additional diagnostic testing, obtaining a venous or central access line, applying oxygen, intubation, use of vasopressors, or supporting cardiopulmonary resuscitation [3, 16, 17]. Interventions were often performed in patients admitted to the ICU in this study because they were more likely to have screened for RRT due to septic shock, respiratory distress, and cardiogenic shock. It is well known that these diseases [18–21] require intensive care because of their high severity and require more intervention.

According to a recent review, RRT interventions have improved patient safety [2, 22]. RRT performance is generally measured in terms of cardiac arrest, unexpected ICU hospitalization, and mortality [23]. Patients with RRT activation tended to have more ICU admissions and a relatively high mortality rate [24]. In this study, the in-hospital mortality rate of RRT-screened patients was 23.5%. The mortality rate has been variously confirmed, ranging from 10.6% to 42.2% [2, 5, 25, 26]. Several studies have shown that RRT interventions reduce mortality in hospitals [2, 3, 22, 27]. Maharaj et al. showed that RRT implementation was associated with an overall hospital mortality reduction in adult patients (relative risk [RR] 0.87, 95% CI 0.81–0.95, *p* < 0.001) and was associated with a reduction in cardiopulmonary arrest in adults (RR 0.65, 95% CI 0.61–0.70, *p* < 0.001) [2]. Chan et. al. showed that RRT activation in adults was associated with a 33.8% reduction in cardiopulmonary arrest rates outside the ICU (RR 0.66, 95% CI, 0.54–0.80) but was not associated with lower hospital mortality rates (RR, 0.96; 95% CI, 0.84–1.09) [27]. However, some studies have shown that RRT interventions do not affect mortality [16]. In the medical early response intervention and therapy study [28], the medical emergency team system did not substantially affect the incidence of cardiac arrest, unplanned ICU admissions, or unexpected death. Therefore, while these results remain controversial, the potential for RRTs to improve meaningful outcomes exists. Therefore, understanding the patient group and the prognosis of patients admitted to the ICU can help improve the effectiveness of RRT.

In this study, higher SOFA score, NEWS, lactate level, and platelet count were factors associated with ICU admission. Higher SOFA score [29–31] and NEWS [14, 32] are well-known factors related to patient severity. Higher scores indicate severe disease in patients; thus, it may have been associated with the patient's ICU admission. Lactate level is known to be related to the severity of systemic hypoperfusion, and high lactate levels are associated with disease severity [33].

Age; screening for medical reasons; higher SOFA score; solid tumor; hematologic malignancy; and higher T-bilirubin, lactate, and CRP levels were associated with in-hospital mortality in this study. Among these factors, higher SOFA scores and lactate levels were also associated with ICU admission. These results were similar to those of other studies. In terms of acute deterioration after more than 7 days of hospitalization, septic shock was an independent risk factor for in-hospital mortality and ICU transfer [1, 34]. Sepsis and septic shock account for a large proportion of ICU admissions and have been shown to be associated with high in-hospital mortality [35–38]. In a study by Shappell et al., among the deceased patients screened by RRT, more patients were older (median age 72 vs. 66 years), were admitted for non-cardiac medical illness (70% vs. 58%), and had a greater median LOS before RRT screening (81 vs. 47 h) [39]. In a study by Lee et al., the presence of malignancy was independently associated with in-hospital mortality [40]. Therefore, more careful treatment when patients with these conditions are screened by RRT may help improve patient prognosis.

This study had several limitations. First, this was a retrospective study performed at a single medical center. Second, the possibility of selection bias cannot be ruled out because the data of many patients are recorded in the EMR by the nurse. However, if the patient had a deteriorating condition, then we trained nurses to check vital signs several times and to input the worst value into the EMR; hence, it was thought that the selection bias might be small. Third, because the RRT was not screened for 24 h, the patient groups that existed at the time when the RRT was not screened were not included in the study. In Fernando's study, patients with acute worsening in the ward assessed by RRT at night (17:00–07:59) had a higher risk of in-hospital death. However, daytime RRT activation is associated with an increased probability of ICU admission [41]. Therefore, exclusion of patients who deteriorated between 11 PM and 7 AM may have affected in-hospital mortality and ICU admission. However, in this study, RRT was not activated only during a shorter time period (11 PM to 7 AM). As for ICU admission, the impact of time is likely to be small because the intensivists participating in the RRT were also involved in ICU admission even when the RRT was not activated. Fourth, we conducted a study on a group of patients screened for RRT; therefore, we could not obtain data on patients who were not screened for RRT in the ward and who were transferred to the ICU.

## Conclusions

In this study, 35.5% of patients screened by RRT were admitted to the ICU. Factors associated with ICU admission were higher SOFA score, NEWS, platelet count, and lactate level. Therefore, close monitoring and transfer to the ICU should be considered when these patients are screened using RRT.

## Data Availability

The datasets used and/or analyzed during the current study are available from the corresponding author upon reasonable request.

## Abbreviations

RRT: Rapid response team
ICU: Intensive care unit
DNR: Do not resuscitate
EMR: Electronic medical record
NEWS: National Early Warning Score
SOFA: Sequential Organ Failure Assessment
IRB: Institutional review board
OR: Odds ratio
CI: Confidence interval
CRP: C-reactive protein
LOS: Length of stay
RR: Relative risk
